# Relevance of the Pyroptosis-Related Inflammasome Pathway in the Pathogenesis of Diabetic Kidney Disease

**DOI:** 10.3389/fimmu.2021.603416

**Published:** 2021-02-22

**Authors:** Pan Liu, Zhengdong Zhang, Yao Li

**Affiliations:** ^1^ Department of Endocrinology, The First Affiliated Hospital of Chengdu Medical College, Chengdu, China; ^2^ Department of Orthopedics, The First Affiliated Hospital of Chengdu Medical College, Chengdu, China

**Keywords:** pyroptosis-related, inflammasome pathway, pathogenesis, diabetic kidney disease, targeted inhibition

## Abstract

Diabetic kidney disease (DKD) is a major cause of chronic kidney disease (CKD) in many developed and developing countries. Pyroptosis is a recently discovered form of programmed cell death (PCD). With progress in research on DKD, researchers have become increasingly interested in elucidating the role of pyroptosis in DKD pathogenesis. This review focuses on the three pathways of pyroptosis generation: the canonical inflammasome, non-canonical inflammasome, and caspase-3-mediated inflammasome pathways. The molecular and pathophysiological mechanisms of the pyroptosis-related inflammasome pathway in the development of DKD are summarized. Activation of the diabetes-mediated pyroptosis-related inflammasomes, such as nucleotide-binding oligomerization domain-like receptor protein 3 (NLRP3), Toll-like receptor 4 (TLR4), caspase-1, interleukin (IL)-1β, and the IL-18 axis, plays an essential role in DKD lesions. By inhibiting activation of the TLR4 and NLRP3 inflammasomes, the production of caspase-1, IL-1β, and IL-18 is inhibited, thereby improving the pathological changes associated with DKD. Studies using high-glucose–induced cell models, high-fat diet/streptozotocin-induced DKD animal models, and human biopsies will help determine the spatial and temporal expression of DKD inflammatory components. Recent studies have confirmed the relationship between the pyroptosis-related inflammasome pathway and kidney disease. However, these studies are relatively superficial at present, and the mechanism needs further elucidation. Linking these findings with disease activity and prognosis would provide new ideas for DKD research.

## Introduction

Cell death includes pyroptosis, apoptosis, necroptosis, necrosis, and autophagy, depending on different biochemical mechanisms and signal transduction pathways (1–5). In 2001, Cookson et al. (6) described a form of cell death in macrophages that depends on caspase-1, which was accompanied by the release of many pro-inflammatory factors. The term “pyroptosis” comes from the Greek “pyro” meaning fire or fever and “ptosis” meaning falling, to describe the pro-inflammatory properties of this cell death process and its relationship with the release of mature interleukin (IL)-1β and IL-18. Pyroptosis is a form of inflammation that is activated by bacteria, pathogens, or their endotoxins, leading to the subsequent activation of caspase-1, accompanied by cell swelling, cell membrane pore formation, cell membrane rupture, cell-permeable dissolution, DNA lysis, inflammasome activation, as well as the release of cell contents and inflammatory mediators, resulting in a robust inflammatory response. This response leads to programmed cell death (PCD) (7, 8). The immune response of the innate immune system after pathogen invasion plays a vital role in maintaining tissue homeostasis and the immune response. Pyroptosis is involved in the innate immune response and protects the body from infection by pathogenic microorganisms (9–11); however, excessive pyroptosis can lead to a variety of autoinflammatory and immune diseases such as massive cell death, tissue damage, organ failure, and even septic shock (12, 13). Recent studies have shown that scorch death plays a vital role in diseases such as liver disease (14), atherosclerosis (15), diabetes mellitus (16), gout (17), epilepsy (18), and tumors (19, 20).

Diabetes is a major global public health problem. The number of people with diabetes increased from 108 million in 1980 to 422 million in 2014 (21), and diabetes is among the leading causes of kidney failure. Diabetic kidney disease (DKD) causes glomerular hypertrophy, basal membrane thickening, glomerular sclerosis, Kimmelstiel-Wilson nodules, an increased glomerular filtration rate (GFR), clinical proteinuria, hypertension, and edema, and is often associated with diabetic retinopathy (22, 23). DKD leads to a decline in the quality of life and shortening of the survival time of patients, thus leading to heavy social and economic burdens. Fernández-Real (24) reported that innate immunity is related to the production of inflammatory cells, and development of obesity, insulin resistance, and other diabetic complications. Microinflammation and extracellular matrix amplification are common pathways for the progression of DKD. Various molecules associated with the inflammatory pathway in DKD include proinflammatory cytokines, chemokines, and Toll-like receptors (TLRs). As an essential innate immune response in the body, pyroptosis is closely related to the progression of DKD owing to the involvement of various pro-inflammatory factors in its activation pathway.

This review focuses on the three pathways of pyroptosis generation. The molecular and pathophysiological mechanisms of pyroptosis-related inflammasomes pathway in the development of DKD are summarized. With this review, we attempted to provide new insights for researchers regarding the development of potential therapies for DKD.

## Enzymes and Proteins Associated With Pyroptosis

### Caspase Family

Caspase is a family of cysteine proteases with a primary function in mediating cell death, including apoptosis and pyroptosis (25). Caspase plays a vital role in embryonic development and in maintaining adult tissue balance. The sub-members of this family include caspase-1, caspase-11, and caspase-12 from mouse sources; and caspase-1, caspase-4, caspase-5, and caspase-12 of human origin. Their common features are control of the inflammatory response of host cells to pathogen invasion and the stimulation of damage by the host cell cytoplasm. Pro-caspases are inactive monomers that are not activated until they are subjected to specific stimuli (26). Pro-caspases become activated once they are absorbed into the multi-protein complex of the inflammasome, and then cleave the inactive pro-IL-1β and pro-IL-18 into active IL-1β and IL-18 during the pyroptosis process (27, 28). Caspase-11 can be directly activated by sensing lipopolysaccharides (LPS) in cells infected by various Gram-negative bacteria, thereby inducing pyroptosis in macrophages (7, 29). Similar to caspase-11, caspase-4 and caspase-5 both induce pyroptosis during LPS sensing (30). Caspase-8 has always been considered an apoptosis-related caspase. Subsequent studies showed that caspase-8 can directly regulate the cleavage and activation of gasdermin proteins under specific conditions to induce pyroptosis (31, 32). However, caspase-8 has significantly weaker processing power on gasdermin D (GSDMD) than caspase-1 (33). Recent studies have found that caspase-8 may be an important molecular switch that controls apoptosis, necroptosis, and pyrolysis, and prevents tissue damage (34, 35). Caspase-12 is also an inflammatory caspase, although its function is unknown (36).

### Gasdermin Family

Six members of the gasdermin family have been identified in humans: gasdermin A (GSDMA), gasdermin B (GSDMB), gasdermin C (GSDMC), GSDMD, gasdermin E (GSDME, also known as DFNA5), and pejva-kin (PJVK, also known as DFNB59). Seven members of the gasdermin family have been identified in mice: GSDMAs (GSDMA1–3) and GSDMCs (GSDMC1–4) (37–39). The entire gasdermin family has a common membrane-targeting mechanism (40). Among them, GSDMD is currently the gasdermin protein that has been most strongly associated with pyroptosis. The N-terminal and C-terminal domains are formed after cleavage by caspase. The N-terminal domain can be connected to phosphatidylinositol, phosphatidic acid, and phosphatidylserine on the cell membrane, resulting in their aggregation and insertion into the cell membrane to form membrane-spanning pores. GSDM pores are large, non-selective pores with an external diameter of 32 nm and an internal diameter of 10–20 nm (41, 42). This pore size is sufficient to allow for the inflow of H_2_O and Ca^2+^ to cause cell swelling. When the cell swells to a certain degree, it disintegrates. The cell contents such as K^+^, IL-1β, IL-18 (about 4–8 nm in diameter), and other small cytosolic proteins flow out, ultimately resulting in pyroptosis (43–49). The C-terminal domain (GSDMD-CT) is removed, and it is hypothesized to fold back on the N-terminal domain of gasdermin (GSDMD-NT) to inhibit N-terminal function, thereby inhibiting the formation of cell membrane pores and blocking the process of pyroptosis (42, 43). The pore-forming activity of GSDMD plays a vital role in the downstream pathway of pyroptosis mediated by inflammatory caspase (50). GSDMD proteins have been reported to independently modulate inflammatory mediators such as IL-1β release and cell membrane breakdown (48). Given the above characteristics, GSDMD is known as the executor of cell pyroptosis. In addition to GSDMD, mutations in the hydrophobic core of the C-terminal of GSDMA, GSDMA3, GSDMC, and GSDME can cause pyroptosis (50). For example, caspase-3 releases the N-terminal through proteolytic cleavage of GSDME, forming a hole in the membrane, and then converts apoptosis induced by tumor necrosis factor (TNF) or chemotherapy into pyrolysis (51–53). Gasdermin family members are widely expressed in different cells and tissues, but are mainly found in the gastrointestinal tract, skin, and immune cells, indicating that they play an essential role in the physical and mucosal barrier system, and actively eliminate infected cells through pyroptosis (38, 54). Recent studies have suggested that GSDMD pores formed on the plasma membrane can enable Ca^2+^ influx and activate the endosomal sorting complexes required for transport (ESCRT) mechanism to initiate the repair of membrane pores (55, 56). However, the specific detailed mechanism requires further study and clarification.

## Innate Immune Pattern-Recognition Receptors (PRRs) Related to Pyroptosis

The PRR family includes TLRs, C-type lectin receptors (CLRs), retinoic acid-induced gene protein I (retinoic acid-induced gene)-like receptors (RLRs), and nucleotide-binding oligomerization domain-like receptors (NLRs). Some PRRs can form oligomeric protein structures called inflammasomes, promote the protein maturation of IL-1 family cytokines (i.e., IL-1 and IL-18), and mediate pyroptosis in inflammatory forms, accompanied by the final secretion of downstream mediators of inflammation, thereby mobilizing the recruitment of a large number of host immune cells that have different immune outcomes and promote acute inflammatory processes (40, 57). We summarize two representative receptors in this section: TLRs and nucleotide oligomerization domain (NOD)-like receptors (NLRs).

### TLRs

TLRs are the first family of innate immune receptors, described as type I transmembrane proteins anchored to the plasma membrane or endolysin membrane. The primary role of TLRs is to recognize the PRR-mediated activation of pathogen-associated molecular patterns (PAMPs) and host damage-associated molecular patterns (DAMPs), and to induce pyroptosis as a response. More than 10 TLRs have been identified in humans and 12 TLRs have been identified in mice (58). TLRs on the cell surface can recognize extracellular pathogens, whereas microbial nucleic acids are sensed by TLRs located in lysosomes. The changes in 1eucine-richrepeats (LRRs) of different TLRs provide specificity for the ligands that they can recognize. Ligand binding causes most TLRs to form dimers. In addition to forming homodimers, some TLRs can also recognize other additional ligands by forming heterodimers, co-receptors, or accessory proteins. In the presence of ligands, TLR dimers undergo conformational changes such that the intracellular TLR domain can join the downstream signal transduction pathway for adaptor initiation (59, 60).

### NLRs

NLRs are the largest family of cytoplasmic receptors and have a common central NOD. The NLR family is divided into four subfamilies: NLRAs, NLRBs, NLRCs (including NOD1, NOD2, NLRC3, NLRC4, and NLRC5), and NLRPs (including NLRP1–14). NLRs have a variety of immune functions, including response to infection and the formation of inflammasomes (NLRP1, NLRP3, and NLRC4) (61, 62), regulation of antigen presentation (NLRC5, CIITA) (63, 64), regulation of homeostasis in microbial clusters (NLRP6), and a regulatory role in the responses of nuclear factor kappa B (NF-кB) (NLRP6, NLRP12, and NLRC3), MAVS (NLRX1), and STING (NLRC3, NLRX1) (65–68). NAIP/NLRC4 oligomeric polymers rely on the CARD-CARD interaction to recruit apoptosis-associated speck-like protein containing CARD (ASC), and then activate caspase-1 and downstream effector functions. PRR activation (especially those in the cytoplasm), in addition to the induction of cytokine transcription, can also induce pyroptosis and stimulate inflammatory responses. Intracellular bacteria are killed directly by destroying the replicative niche of pathogens and intracellular traps induced by holes, thereby enhancing the immune defense function of pyroptosis (9, 11). NLRP3 is a key member of the NOD-like receptor family, which recognizes microbial and non-microbial risk signals, and induces aseptic inflammation under different conditions (69). In the pyroptosis pathway, NLRP3 can be combined with ASC to recruit pro-caspase-1 to form inflammasomes, which are converted into caspase-1 by hydrolysis. Previous studies have suggested that NOD2, TLR2, TLR4, and NLRP3 inflammasome-mediated inflammation participate in the persistence of DKD inflammation (70, 71).

## Molecular Mechanism of Pyroptosis

### Canonical Inflammasome Pathway Associated With Pyroptosis

Cells are stimulated by signals from bacteria and viruses, and different cytoplasmic sensor proteins trigger responses to pathogens and inflammatory factors. Dimerization occurs through combination of the adaptor protein ASC or NLRC4 with the pro-caspase-1 monomer, which activates pro-caspase-1 to become mature caspase-1. Meanwhile, caspase-1 cleaves GSDMD and activates the inactive precursor IL-1β into mature IL-1β. After GSDMD is cleaved, the domains at both ends of the NC are separated, and GSDMD-NT is released. The released GSDMD-NT forms a pore in the cell membrane by recognizing and binding phospholipid molecules on the cell membrane. The formation of pores destroys cell potential energy penetration, leading to cell swelling and eventually cell pyroptosis. IL-1β is also released from cells through the pores, causing a robust inflammatory response (43, 72, 73).

### Non-canonical Inflammasome Pathways Associated With Pyroptosis

In 2011, Kayagaki et al. (74) discovered non-canonical pyroptotic pathways. In contrast to canonical pyroptotic pathways, the cell wall LPS of Gram-negative bacteria bypasses TLR4 and directly combines with the pro-caspase (-4 and -5 in humans and -11 in murine) to form activated caspase-4/5/11. The activated caspase-4/5/11 cleaves GSDMD and promotes the activation of pro-IL-1β and pro-IL-18 into mature IL-1β and IL-18. Similarly, GSDMD-NT forms a hole in the cell membrane, which causes the release of IL-1β and IL-18 in the cell and induces pyroptosis (29, 38, 75). This pathway does not involve caspase-1; in the absence of caspase-1, human caspase-4/5 and murine caspase-11 can also induce pyroptosis with all associated morphological characteristics (40).

From the perspective of these two inflammasome pathways associated with pyroptosis, in the canonical inflammasome pathway, inflammation sensors detect different microbial signals and activate caspase-1 through ASC or NLRC adaptors. In contrast, the non-canonical inflammasome pathway is activated by caspase-4, caspase-5, and caspase-11, which are directly combined with LPS (76). In addition, recent studies have shown that caspase-11 directly binds to *Leishmania* lipophosphoglycan (LPG) (77) and oxidized phospholipids (oxPAPC) (78). Although the activation pathways are different, the downstream signaling pathways are all activated caspases that cleave GSDMD and release the N-terminal domain to form membrane pores, eventually leading to pyroptosis. In other words, GSDMD is a necessary downstream component of both the canonical and non-canonical inflammasome pathways associated with pyroptosis (41–43, 46, 47).

### Caspase-3-Mediated Inflammasome Pathway Associated With Pyroptosis

**Figure 1:**
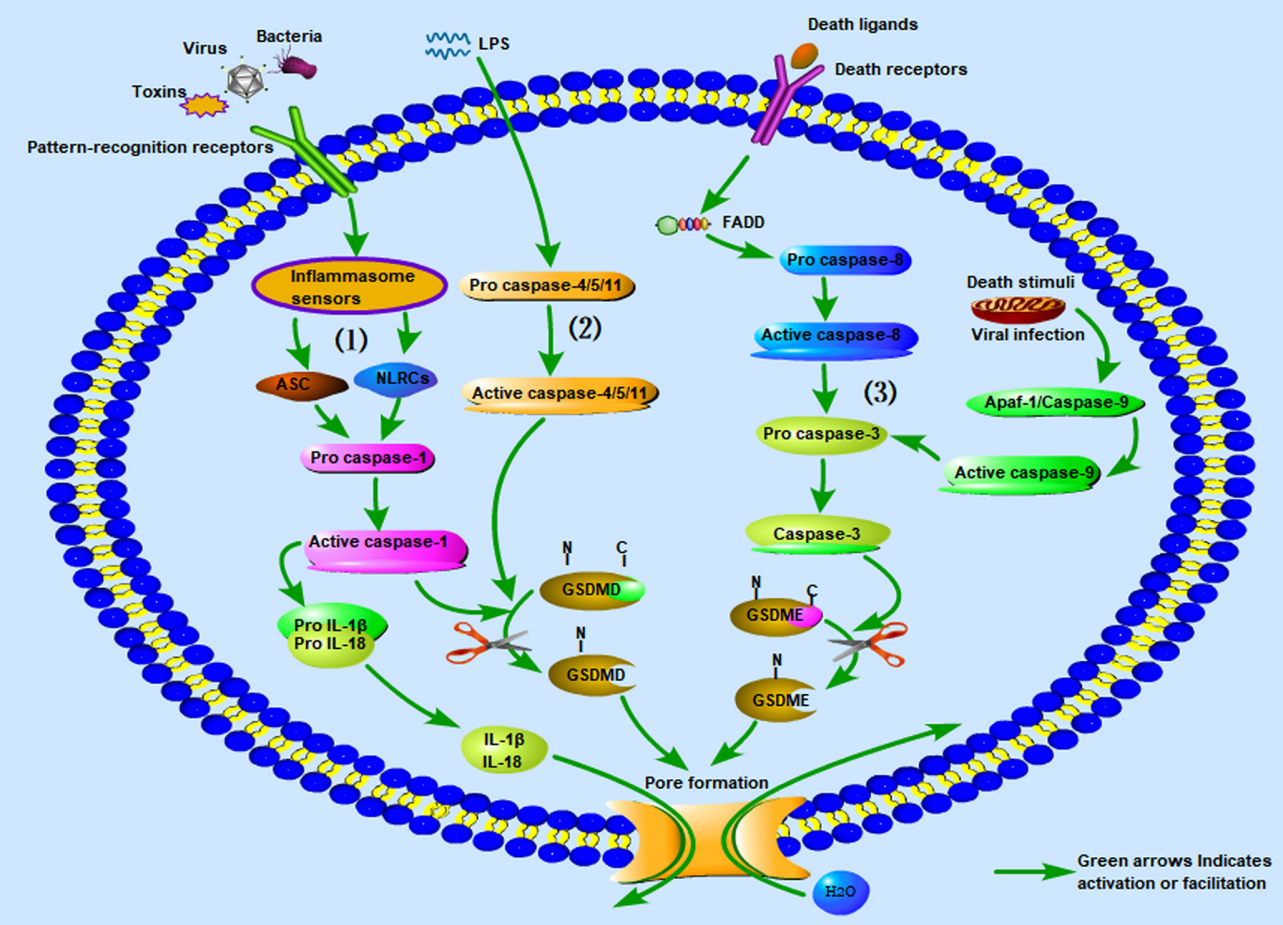
The three kinds of inflammasome pathways associated with pyroptosis. (1) Canonical inflammasome pathway associated with pyroptosis; (2) non-canonical inflammasome pathway associated with pyroptosis; (3) caspase-3 mediated inflammasome pathway associated with pyroptosis. LPS, Lipopolysaccharide.

In addition, a new pyroptosis pathway was recently discovered. Caspase-3 is well-known as an important effector associated with apoptosis (79). Previous studies have suggested that caspase-3 is not involved in pyroptosis (80). However, researchers recently found that GSDME can convert caspase-3 induced apoptosis into pyroptosis through TNF-α and some chemotherapeutic drugs (52). Various death stimuli or viral infections can lead to an increase in the permeability of the outer mitochondrial membrane, causing the release of cytochrome C and binding to apaf-1, thereby enabling the assembly of apaf-1 apoptotic bodies and activation of caspase-9. The active caspase-9 then cleaves pro-caspase-3 to generate the active caspase-3 heterodimer. In addition, caspase-3 can be activated through the death receptor pathway, which is itself activated by the death receptor ligand on the cell membrane, and then pro-caspase-8 is activated to caspase-8. Active caspase-8 cleaves pro-caspase-3 to generate the active caspase-3 heterodimer (51). Caspase-3 cleaves GSDME into the N-terminal fragment of GSDME (GSDME-NT) and the C-terminal fragment of GSDME (GSDME-CT). GSDME-NT forms membrane pores on the cell membrane and induces pyroptosis (20, 52). In the course of this pathway, although the apoptosis-related proteins in the cells are activated at nearly the same time, the process of cell pyroptosis is faster; therefore, the cells eventually appear as pyroptotic (52). For a more intuitive understanding, we have summarized the three kinds of inflammasome pathways associated with pyroptosis along with graphical interpretations in **Figure 1**.

## Correlation of the Pyroptosis-Related Inflammasome Pathway With DKD

Some recent studies have demonstrated that IL-1β, an inflammatory factor released by cells during pyroptosis, plays an important role in the pathogenesis of type 2 diabetes mellitus (T2DM) (81). Increased IL-1β has emerged as an essential factor for predicting the occurrence of T2DM. DKD is a common clinical complication in patients with diabetes, and is the leading cause of chronic kidney disease (CKD) in many developed and developing regions. Persistent aseptic inflammatory reactions in the kidney tissue are the pathophysiological basis of diabetic nephropathy (DN) that lead to glomerular capillary damage. The clinical features of DN are a gradual decline in renal function, abnormal levels of albumin (microalbuminuria) in the urine (30 mg/day or 20 g/min), and subsequent proteinuria and end-stage renal disease (ESRD) (82, 83). Once ESRD develops, the mortality rate is high, representing a critical clinical issue (84, 85).

### Reactive Oxygen Species (ROS)/Thioredoxin-Interacting Protein (TXNIP)/NLRP3 Inflammasome Signaling Pathway

With the expansion of research on DKD, there has been more interest on the potential role of pyroptosis in DKD pathogenesis. The hyperglycemia associated with DN is due to insufficient insulin secretion or insulin resistance, which produces hypoxia and causes excessive production of inflammatory cytokines. If oxidative stress reactions persist, many inflammatory cells are immersed in the matrix (86). The excessive activation of inflammatory cytokines can promote the progression of renal fibrosis (87, 88). Numerous studies have shown that activation of inflammatory factors caused by hyperglycemia plays a crucial role in the progression of DKD (89–91).

The NLRP3 inflammasome is involved in the pathogenesis of various kidney diseases, including acute kidney injury, CKD, DKD, and crystal-related nephropathy (92, 93). The NLRP3 inflammasome promotes disease occurrence and progression in DKD in a high-glucose environment. The production of mitochondrial ROS has been shown to initiate the activation of NLRP3 inflammasomes under diabetic conditions (94–96), further establishing a causal relationship between NLRP3 inflammasome activation and DKD. Inhibition of NLRP3 in the kidney (*via* silencing of NLRP3 or the studies in NLRP3 knockout mice) may improve renal function, and attenuate glomerular hypertrophy, glomerulosclerosis, mesangial expansion, interstitial fibrosis, inflammation, and expression of TGF-β1 and connective tissue growth factor (CTGF). Inhibition of NLRP3 or caspase-1 inflammasome activation, thereby inhibiting renal inflammation and fibrosis (at least in part), *via* suppression of oxidative stress in DN imparts protective effects on the kidney (97–99). By inhibiting NLRP3 upstream of the pyrolysis-related inflammasome pathway, the downstream expression of caspase-1, IL-1, and IL-18 can be progressively inhibited. Notably, the NLRP3 inflammasome can be activated *via* the canonical and non-canonical inflammasome pathways associated with pyroptosis (100). These findings provide a solid theoretical basis for how the NLRP3 inflammasome can be activated by inducing caspase-1 as an essential mediator of pro-inflammatory cytokine production (101, 102).

**Figure 2:**
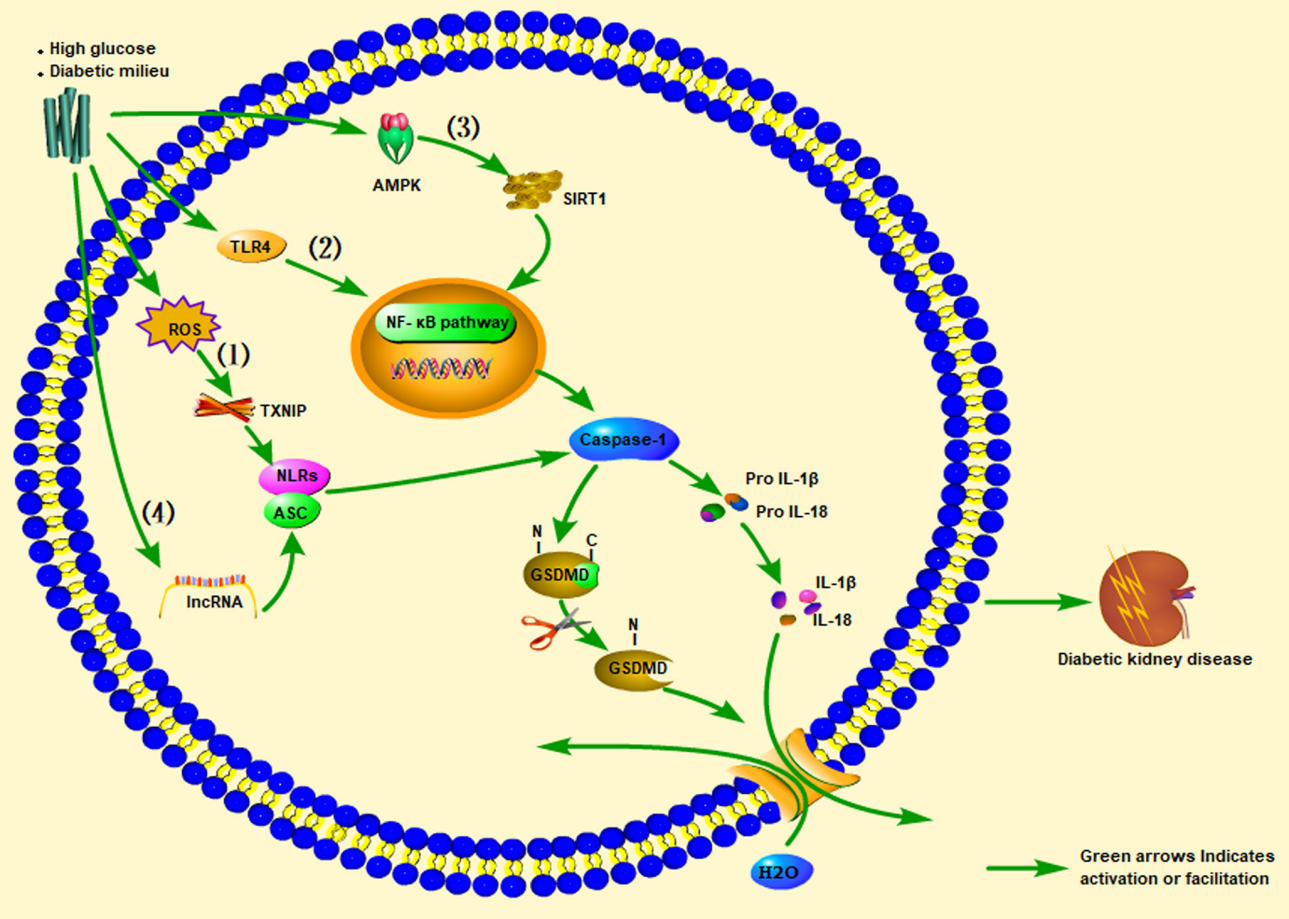
Promotion of inflammasome pathways associated with pyroptosis of cells in the kidney (glomerular endothelial cells, tubular epithelial cells, podocytes, tubular epithelial cells) under high-sugar or diabetes conditions, leading to diabetic kidney disease (DKD). In renal cells stimulated by high glucose (HG): (1) ROS/TXNIP/NLRP3 inflammasome signaling pathway, (2) TLR4/NF-κB inflammasome signaling pathway, (3) AMPK/SIRT1/NF-κB inflammasome signaling pathway, or (4) lncRNA)-related signaling pathways, they all activate pro-caspase-1 to become mature caspase-1. Caspase-1 cleaves GSDMD and activates the inactive pro IL-1β and pro-IL-18 to become mature IL-1β and IL-18, and the released GSDMD-NT forms a pore in the cell membrane, ultimately leading to DKD. ROS, Reactive oxygen species; TXNIP, Thioredoxin-interacting protein; AMPK, Adenosine 5’-monophosphate (AMP)-activated protein kinase; SIRT1, Silent information regulation 2 homolog1; NF-κB, Nuclear factor kappa-B.

Clinical studies have found that compared with diabetic and non-diabetic patients without proteinuria, diabetic patients with proteinuria have significantly higher expression levels of IL-1β, IL-18, NLRP3, and serum IL-1β and IL-18 levels, with a proteinuria-positive correlation (95, 102, 103). Clinical research results also show that inhibiting IL-1β can prevent the progression of T2DM (104). ASC and caspase-1 were found to be highly expressed in a streptozotocin (STZ)-induced DN rat model, accompanied by hyperuricemia and hyperlipidemia, and IL-1β and IL-18 levels were elevated. The NLRP3 inflammasome-caspase-l-IL-1β/IL-18 axis is considered to play a critical role in DKD. Likewise, animal experiments have found that uric acid-lowering drugs (such as allopurinol and quercetin) can reduce uric acid and blood lipid levels, inhibit the activation of NLRP3 inflammasomes, and prevent kidney damage caused by STZ (105). Another study found that significant inhibition of NF-κB, and the decrease in IL-1β and TNF-α levels in diabetic rats were related to reductions in TXNIP and NLRP3 expression levels in diabetic kidneys (106). A clinical study showed that IL-18 levels in the serum and urine of patients with T2DM were positively correlated with the degree of proteinuria during follow-up, indicating that IL-18 may also be a risk factor for DKD (107). Insulin secretion disorders caused by pancreatic β-cell dysfunction and impaired insulin action caused by enhanced insulin resistance lead to hyperglycemia in T2DM (108). The combination of guava (*Psidium guajava*), with demonstrated antioxidant and anti-inflammatory effects, and trehalose on protecting the kidney and pancreas from damage was explored in a rat model of T2DM. The results showed that guava juice and trehalose could inhibit the secretion of IL-1β in the pancreas and kidneys caused by diabetes, and could prevent apoptosis and pyroptosis (109). An et al. (110) administered punicalagin to a mouse model of DN induced by a high-fat diet (HFD) and STZ. After punicalagin intervention, blood urea nitrogen (BUN), serum creatinine (CREA), and urine albumin-creatinine ratios (UACR) were significantly reduced, and the glomerular interstitial hyperplasia and glomerular hypertrophy scores were reduced. This treatment also reduced the expression levels of IL-1β, caspase-1, GSDMD, and NLRP3. The authors also found that punicalagin reduced the high-glucose–mediated protein expression of nicotinamide adenine dinucleotide phosphate (NADPH) oxidase 4 (NOX4) and reduced mitochondrial damage. Thus, by regulating activation of the NLRP inflammasome, the expression of caspase-1 increases, promoting the maturation and release of IL-1β and IL-18, thereby continuing to produce inflammation that leads to kidney damage (**Figure 2**).

Previous studies have found that the A1 adenosine receptor (A1AR) is widely distributed in the renal peritubular capillaries (PTCs) and glomerular afferent arterioles. A1AR is considered an essential regulator of renal tubular-glomerular feedback (TGF) (111). Knockout of A1AR in mice aggravated proteinuria and glomerular damage (112). Moreover, a recent study showed that the A1AR agonist 2-chloro-N6 cyclopentyladenosine (CCPA) plays a protective role in albuminuria related to the loss of megalin in the proximal renal tubules by inhibiting caspase-1/IL-18 signaling in DKD (113).

### TLR4/NF-κB Inflammasome Signaling Pathway

TLR4 usually signals through its downstream partner MyD88 to activate the NF-κB pathway, leading to ROS and cytokine production. In podocytes stimulated by high glucose, TLR4 activates NF-κB, and increases the release of pro-inflammatory cytokines and chemokines (**Figure 2**) (114, 115). Wang et al. (116) found that renal tubular damage in DKD patients upregulated the expression of TLR4 and GSDMD in the kidney tissue. In the paraffin-embedded sections of human DN tissues, immunohistochemical staining showed that the expression of GSDMD and TLR4 was positively correlated with albuminuria, interstitial fibrosis, and tubular atrophy scores, and was negatively correlated with the estimated glomerular filtration rate. Injection of the TLR4 inhibitor TAK-242 to db/db mice improved the brush border peeling and atrophy of the kidney tubules, along with interstitial fibrosis. Simultaneously, TLR4 inhibitors could reduce the expression levels of GSDMD and IL-18 in the renal tubular cells of db/db mice, the protein levels of caspase-1 and GSDMD-NT in the renal cortex tissue, and the level of IL-1β in renal homogenates. In addition, human renal tubular epithelial (HK-2) cells treated with high glucose and TAK-242 or parthenolide (an inhibitor of NF-κB) yielded similar results in western blotting, enzyme-linked immunosorbent assay, and flow cytometry. Studies with a diabetic mouse model and HK-2 cell experiments showed that inhibiting TLR4/NF-κB signaling can reverse the increase in GSDMD-NT expression in a high-glucose environment while inhibiting the release of IL-1β. This finding indicates that TLR4 inhibitors significantly inhibited GSDMD-related pyroptosis and reduced kidney damage in db/db mice. In addition, the proteinuria, renal insufficiency, inflammation, and renal fibrosis of STZ-induced diabetic mice with TLR4 knockout were protected, and TLR4 inhibition prevented renal tubular damage and reduced the loss of podocytes in DKD (117, 118). Therefore, the TLR4/NF-κB signaling pathway is involved in the expression of GSDMD in DKD (116).

### AMPK/SIRT1/NF-κB Inflammasome Signaling Pathway

Li et al. (119) found that geniposide can alleviate renal dysfunction in DN mice induced by an HFD and STZ treatment, which is manifested by reduced serum creatinine SCr) BUN, TNF-α, IL-6, and IL-1β levels. Histological examinations showed that geniposide could reduce glomerular basement membrane thickening and inflammatory cell infiltration. Geniposide also reversed the significant decrease in AMPK, p-AMPK, and SIRT1 levels in a podocyte model induced by high glucose. Geniposide effectively blocked oxidative stress and inflammation, thereby inhibiting DN development. The mechanism was suggested to involve the APMK/SIRT1/NF-κB pathway. Similarly, Chen et al. (120) reported that catalpol could effectively inhibit oxidative stress and inflammation in HFD/STZ-induced DN mice and in a high-glucose–induced podocyte model, and that the mechanism may be related to the AMPK/SIRT1/NF-κB pathway, indicating that catalpol has potential value in the treatment of DN.

### Long Non-coding RNA (lncRNA)-Related Signaling Pathways

With recent research on lncRNAs, exploration of the roles of non-coding RNAs in DN has intensified. Based on circRNA microarray analysis in glucose-stressed HK-2 cells, circACTR2 was found to regulate the pyroptosis and fibrosis of proximal renal tubular cells induced by high glucose. Knockout of circACTR2 significantly inhibited high-glucose–induced IL-1β release, and the production of collagen IV and fibronectin in HK-2 cells. By clarifying the role of circRNAs in the renal tubular cell pyroptosis-related inflammasome, new insights into the pathogenesis and treatment strategies of DKD may be attained (121). Li et al. (122) discovered that miR-23c, as a target of metastasis-associated lung adenocarcinoma tran 1 (*MALAT1*), directly inhibits the expression of embryonic lethal, abnormal vision, Drosophila-like 1 (*ELAVL1*), thereby reducing the expression of the downstream factors NLRP3, caspase-1, and IL-1β. In addition, silencing KCNQ1OT1 was shown to inhibit high-glucose–induced inflammation, oxidative stress, and pyroptosis in HK-2 cells by upregulating the expression of miR-506-3p (123). Considering that miR-452-5p is a potential target of GAS5, overexpression of GAS5 could downregulate the expression of mir-452-5p, thereby inhibiting NLRP3, caspase-1, IL-1β, and GSDMD-NT expression in high-glucose–induced HK-2 cells (**Figure 2**) (124).

Overall, the studies highlighted above found that upregulation of pyroptosis-related inflammatory factors was associated with DKD. Although angiotensin-converting enzyme inhibitors (ACEIs) or angiotensin II receptor blockers (ARBs) is often used to treat DKD clinically, it cannot reverse the condition of DKD. Therefore, more and more molecular studies on DKD are being conducted to seek for better methods to treat DKD. These researches are summarized in **Table 1**. However, the evidence obtained to date is not sufficient to clarify whether there is a critical relationship between DKD and cell death, and the underlying mechanism of this association. Thus, the key discovery of the possible underlying mechanism linking inflammatory factors and pyroptosis in DKD will provide new insights to clarify and control the incidence and progression of DKD.

**Table 1:** Strategies to inhibit the inflammasome pathways associated with pyroptosis of diabetic kidney disease.

Medication or treatment	Effects on renal tissues	Reference
Quercetin and allopurinol	Inhibiting caspase-1/IL-1β/IL-18 axis	(105)
Cepharanthine and piperine	Inhibiting NF-κB and NLRP3 inflammasome activation	(106)
Guava and trehalose	Inhibiting IL-1β	(109)
Punicalagin	Inhibiting TXNIP/NLRP3 signaling	(110)
CCPA (A1AR agonist)	Inhibiting caspase-1/IL-18	(113)
TAK-242	Inhibiting TLR4/NF-κB signaling	(116)
Parthenolide	Inhibiting NF-κB	(116)
Geniposide	Inhibiting AMPK/SIRT1/NF-κB signaling	(119)
Silencing KCNQ1OT1	Upregulating the expression of miR-506-3p	(123)
GAS5	Reducing the expression of miR-452-5p	(124)

These studies do not prove that pyroptosis is necessarily related to DKD and the mechanism needs further elucidation.

## Conclusions

In this review, we have summarized the mechanism of pyroptosis, including the canonical, non-canonical, and caspase-3-mediated inflammasome pathways associated with pyroptosis. Research on the mechanism of pyroptosis regulation in DKD is rapidly emerging in the field of renal disease and immunology. The activation of diabetes-mediated related factors such as TLR4, NLRP3, caspase-1, IL-1β, IL-18, and GSDMD-NT plays a vital role in the pathophysiology of DKD. By inhibiting the activation of TLR4 or the NLRP3 inflammasome and related pathways, caspase-1, IL-1β, IL-18, and GSDMD-NT are inhibited, leading to renal lesions associated with DKD. The inflammasome is involved in the processes of activation, including the activation of caspase-1, which finally orders the terminal core protein GSDMD to perform its perforation effect that causes the release of IL-1B/IL-18. These are essential links in the process of pyroptosis. Recent studies have confirmed the relationship between the pyroptosis-related inflammasome and kidney disease. However, these studies are relatively superficial at present, they do not prove that pyroptosis is necessarily related to DKD and the mechanism needs further elucidation.

The detailed mechanism underlying the function of GSDMD in DKD in the downstream pathway of pyroptosis remains unclear. Small-molecule inhibitors targeting TLR4, NLRP3, and other inflammatory components are potential therapeutic options for DKD. However, there are still many unknown pathways and targets, and corresponding inhibitors, related to the occurrence and development of DKD related to pyroptosis awaiting further exploration. Studies using high-glucose–induced cell models, HFD/STZ-induced DKD animal models, and human DKD patient kidney tissue biopsies will help determine the spatial and temporal expression of DKD inflammatory components, and link these findings with the activity and prognosis of the disease. These insights may provide research ideas for developing new mechanisms, drugs, and technologies for DKD. Based on the current summary, we propose the following research targets.

First, according to molecular mechanisms related to inflammasomes that have been discovered to date, molecular biology methods could be used to further explore the specific mechanisms of caspase, GSDMD, IL-1β, and IL-18 that contribute to DKD, so as to clarify the pathways underlying the role and relationship of pyroptosis-related inflammasomes in DKD.

Second, based on the known signal activation of ROS, TLR4, NLRP3, and lncRNAs, efforts should be made to discover more small molecules or targeted drugs that can regulate the pathways of DKD-related inflammasomes, thereby bringing new methods and hope for the treatment of DKD.

Finally, more attention should be paid to the pathophysiology of DKD, and to understand the possible potential pathways of the pyroptosis-related inflammasome, which can offer new methods and technologies for the clinical treatment of DKD.

## Author Contributions

All authors listed have made a substantial, direct and intellectual contribution to the work, and approved it for publication.

## Funding

This work was supported by the Sichuan Provincial Health and Family Planning Commission (Project No. 18PJ346).

## Conflict of Interest

The authors declare that the research was conducted in the absence of any commercial or financial relationships that could be construed as a potential conflict of interest.
